# FUNCTIONAL OUTCOMES FOLLOWING SURGERY FOR SPASTIC HIP ADDUCTOR MUSCLES IN AMBULATORY AND NON-AMBULATORY ADULTS

**DOI:** 10.2340/jrm.v56.18356

**Published:** 2024-03-22

**Authors:** Daphnée BRUN, Olivier HAMEL, Emmeline MONTANE, Marino SCANDELLA, Evelyne CASTEL-LACANAL, Xavier DE BOISSEZON, Philippe MARQUE, David GASQ, Camille CORMIER

**Affiliations:** 1 Department of Physiological Explorations, University Hospital of Toulouse, Toulouse; 2 Neurosurgery Department, Neurosciences Pole, CAPIO, Clinique des Cèdres, Cornebarrieu; 3 Laboratory of Gait Analysis, University Hospital of Toulouse; 4 Department of Physical and Rehabilitation Medicine, University Hospital of Toulouse; 5 ToNIC (Toulouse NeuroImaging Center), Inserm, University of Toulouse 3, Toulouse, France

**Keywords:** denervation, gait, muscle spasticity, obturator nerve, neurosurgery

## Abstract

**Objective:**

To evaluate functional outcomes of surgery of spastic hip adductor muscles (obturator neurotomy with or without adductor longus tenotomy) in ambulatory and non-ambulatory patients, using preoperatively defined personalized goals.

**Design:**

Retrospective observational descriptive study.

**Patients:**

Twenty-three patients with adductor spasticity who underwent obturator neurotomy between May 2016 and May 2021 at the Clinique des Cèdres, Cornebarrieu, France, were included.

**Methods:**

Postoperative functional results were evaluated in accordance with the Goal Attainment Scaling method. Patients were considered “responders” if their score was ≥ 0. Secondary outcomes included spasticity, strength, hip range of motion and change in ambulatory capacity. When data were available, a comparison of pre- and postoperative 3-dimensional instrumented gait analysis was also performed.

**Results:**

Among the 23 patients only 3 were non-walkers. Seventeen/22 patients achieved their main goal and 14/23 patients achieved all their goals. Results were broadly similar for both walking goals (inter-knee contact, inter-feet contact, fluidity, walking perimeter, toe drag) and non-walking goals (intimacy, transfer, pain, posture, dressing).

**Conclusion:**

Surgery of spastic hip adductor muscles results in functional improvement in ambulation, hygiene, dressing and posture and can be offered to patients with troublesome adductor overactivity. The use of a motor nerve block is recommended to define relevant goals before the surgery.

LAY ABSTRACT

Following injury to the central nervous system, patients may develop overactivity of the hip adductors, leading to discomfort in activities of daily living. This study aimed to assess the impact of surgery of spastic hip adductor muscle, consisting in obturator neurotomy combined or not with adductor longus tenotomy, on activities of daily living such as walking, hygiene, and dressing. The study employed Goal Attainment Scaling to assess personalized functional goals, using medical reports from assessments carried out as close as possible to 6 months after surgery. The majority of patients achieved their objectives, irrespective of ambulatory or non-ambulatory goals. This study emphasizes preoperative motor nerve blocks for goal definition.

In individuals with brain or spinal cord injury, upper motor neurone syndrome can lead to hip adductor muscles spastic overactivity (1), impacting various aspects of daily life, such as hygiene, dressing, sexual intimacy, sitting, transfers, standing and walking (2).

The main adductor muscles are the adductor magnus (AM), adductor longus (AL), adductor brevis (AB) and gracilis (2). Gracilis, AL and AB are innervated by the anterior branch of the obturator nerve. They insert into the ischio-pubic branch and the pubis and participate in hip flexion (3). They activate during the late terminal stance and the swing phase of gait to promote limb advancement (4). In contrast, the AM, innervated by the sciatic nerve and the posterior branch of the obturator nerve (5), participates in hip extension due to its insertion on the posterior portion of the ischio-pubic branch (3). The AM contracts during stance to stabilize the trunk (4).

First-line treatment for focal spastic overactivity is based on chemodenervation with botulinum neurotoxin (BoNT) (6). BoNT injections in hip adductor muscles have shown improvement in functional abilities, including hygiene (7) and ambulation (8). According to Cioncoloni, combining BoNT injections in the adductor muscles with the quadriceps and/or the sural triceps can enhance ambulatory speed (9). An alternative treatment is chemodenervation with phenol, offering longer yet not definitive efficacy, lasting up to 13 months according to Yadav (10). Phenolization has also demonstrated effectiveness in achieving functional goals related to hygiene and ambulation (11–13).

Selective peripheral neurotomy, sometimes combined with tenotomy, is a surgical alternative for reducing spastic overactivity. This technique is frequently performed on the calf muscles involved in equinovarus foot deformity (14) and on the rectus femoris muscle (15). It involves partial sectioning of motor branches of nerves innervating spastic muscles (16).

The first neurotomy was described by Lorenz on the obturator nerve in 1887 (17). Initially, the obturator neurotomy consisted of complete neurotomy of the obturator trunk. This surgery was performed in children to enhance hygiene, prevent hip subluxation-dislocation, improve sitting ability and occasionally enhance ambulatory ability (16, 18, 19). Haftek (20) reported the recovery of ambulatory capacity in some patients using this technique, although specific details about the study population and its functional abilities are not well-documented.

Currently, obturator neurotomy (ON) involves sectioning only the anterior branch of the obturator nerve to preserve hip stability from the AM (21). To our knowledge, only 1 study has focused specifically on ON with this technique in an adult population. This study evaluated the modification of passive hip range of abduction in 6 non-ambulatory subjects with cerebral palsy, including 2 adults (22). More recently, 2 studies evaluated the functional benefits of ON combined with other neurotomies, demonstrating improvements in the ability to sit (23) and walk (24). The first study included 9 non-ambulatory patients: all underwent sciatic nerve neurotomy and 7 had associated ON (23). In the second study of 14 spinal cord injured patients, all underwent tibial nerve neurotomy, with 13 having ON and 2 having hamstring neurotomy (24).

The primary objective of this study was to evaluate the functional benefits of surgery of the spastic hip adductor muscles in both ambulatory and non-ambulatory patients, using preoperatively defined personalized goals. Secondary aims included evaluating analytical modifications of adductor muscles spasticity and strength, kinematic modification during ambulation, and post-operative complications.

## MATERIALS AND METHODS

### Study design

This study was carried out in the Toulouse University Hospital and in the medical centre “Clinique des Cèdres” from October 2020 to September 2022. This observational descriptive study analysed data collected during routine care. According to French ethics and regulatory law (*Public Health Code*), all patients received information about anonymized data collection, and the study was registered in Neurotobt, Toulouse University Hospital (registration number: RnIPH 2020-59) and covered by the MR-004 (National Commission on Informatics and Liberties/Non-interventional Human Subject Research number: 2206723 v 0).

### Population

Patients with upper motor neurone syndrome who underwent ON at the “Clinique des Cèdres”, either combined or not with adductor muscles tenotomy, between May 2016 and May 2021 were enrolled in this study. Exclusion criteria were: age < 18 years at the date of surgery, the absence of assessment at the Toulouse University Hospital, and opposition to data collection.

### Surgical procedure

**Fig. 1:**
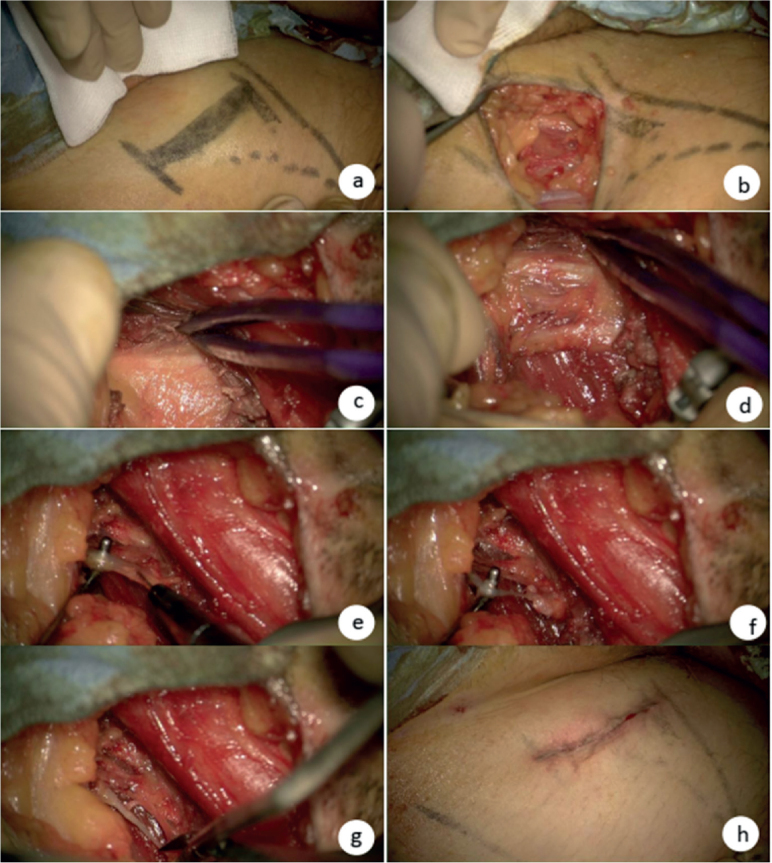
Obturator neurotomy. (a) Incision below the inguinal line. (b) Localization of the saphenous vein. (c and d) The 3 nerve branches of the anterior branch of the obturator nerve before and after resection of the adipose tissue. (e and f) One branch before and after the section at 90%. (g) Resection of the severed nerve. (h) Scar.

Patients were placed in a supine position with abduction and external rotation of both hips. An incision was made over the body of the adductor longus muscle about 3cm below the inguinal line and parallel to it (Fig. 1a). Following the incision, the saphenous vein was identified and positioned laterally (Fig. 1b). The anterior branch of the obturator nerve was dissected into 3 or 4 branches within the adipose tissue (Fig. 1c). After resection of this tissue, electrical stimulation was applied to each branch to trigger contraction of the hip adductor muscles. Each branch was then sectioned until there was no response to 1 mA electrostimulation of the proximal part of the fascicle (24) approximately corresponding to a 90% resection (15) (Fig. 1e and f). To minimize recurrence, portions of severed nerves were resected (Fig. 1g). In the presence of significant retraction of the adductor muscles, a partial tenotomy of AL was conducted.

### Data collection

Preoperative data were retrospectively gathered from reports of different hospitalizations or consultation. In each instance, the preoperative data collected were those closest to the surgical date.

Post-operative data were obtained through post-operative assessments conducted as close as possible to 6 months after surgery. If accessible, early functional results of surgery were also extracted from reports.

Three-dimensional instrumented gait analysis (3D-IGA) data were acquired using an optoelectronic system comprising 8 high-resolution infrared cameras (Vicon, Oxford Metrics, UK). Passive markers were positioned according to the modified Haye model. All 3D-IGAs were performed by the same technician.

### Primary outcome: Goal Attainment Scaling score

The surgical goals were discussed during a medical-surgical concertation a few weeks before the surgery and definitively defined during the pre-operative consultation by a physician with expertise in neuro-orthopaedics and the patient, using the results of the nerve block performed beforehand. These goals were retrieved from reports, identifying a main goal if multiple goals were defined. Postoperative functional outcomes were assessed using the Goal Attainment Scaling (GAS) method. The scale used was from “–3” to “+2”, where “–3” represented worsening; “–2” the initial state, “0” the goal achieved and “+2” the best possible effect that could have been expected. Patients with a GAS-score ≥ 0 were considered “responders”, while those with a GAS-score < 0 were designated “non-responders”.

### Secondary outcomes

Demographic data including age at surgery and the aetiology of spastic paresis, along with the treatment sequence of treatment history (previous surgeries, BoNT injections and anaesthetic motor block) were collected.

Details of post-operative complications and unexpected events, both beneficial or harmful, were documented.

Pre- and post-operative measurements included range of motion, spasticity and strength. All evaluators underwent training by 2 physicians with expertise in neuro-orthopaedics, ensuring standardized assessments. The measurements included intercondylar distance (in centimetres); passive range of abduction (in degrees); adductor spasticity with the Modified Tardieu Scale (MTS) (25, 26) and the Modified Ashworth Scale (5-point scale) (MAS) (25, 27); and adductor strength with the Held score (26). Measurements of the passive range of abduction were conducted as follows: hips and knees extended at 0° for evaluation of the gracilis; hips and knees flexed at 90° for evaluation of the AB and AL; and hips extended at 0° and knees flexed at 90° for evaluation of the AM. Hip flexion strength was assessed in the supine position.

Gait performance was evaluated using the modified Functional Ambulation Classification (mFAC) (28) and by the use of a cane or a walker. To assess ambulation quality, patients were interviewed by the physician before and after surgery. They self-reported their maximum walking distance and frequency of falls using categories such as “exceptional”, “monthly”, “weekly” and “daily”.

For patients who underwent 3D-IGA before and after surgery, 6 variables of interest were recovered: “means of speed”; “step width”; “internal hip rotation at initial contact”; “internal foot progression angle at mid stance”; “maximal thigh flexion”; and “abduction during swing”. Thigh flexion and abduction were represented by the angle between the thigh and the vertical axis in the sagittal and frontal planes, respectively.

### Statistical analysis

A χ^2^ test was performed to compare the proportions of responders between ambulatory and non-ambulatory goals (significant if *p*-value < 0.05).

Comparison of pre- and post-operative range of motion, spasticity and strength was performed with a Wilcoxon test for paired data.

Pre- and post-operative differences for kinematic data were compared at intra-subject level with a Wilcoxon test for paired data. For each outcome, the pre-post difference was considered significant if the *p*-value was < 0.05 and clinically relevant if the pre-post difference was superior to the measurement error of this outcome, i.e. the minimal detectable change (MDC). To determine the MDC, we relied on the different studies cited in McGinley’s systematic review (29). The median value of the MDC was computed from available data, resulting in an MDC of 4° for thigh flexion, 2° for thigh abduction, 3° for foot progression angle, and 6° for hip rotation. When MDC was not available for a parameter, we considered 2 standard deviations (SD) as the measurement error to be exceeded to consider the change in the initial value as clinically relevant.

## RESULTS

**Fig. 2:**
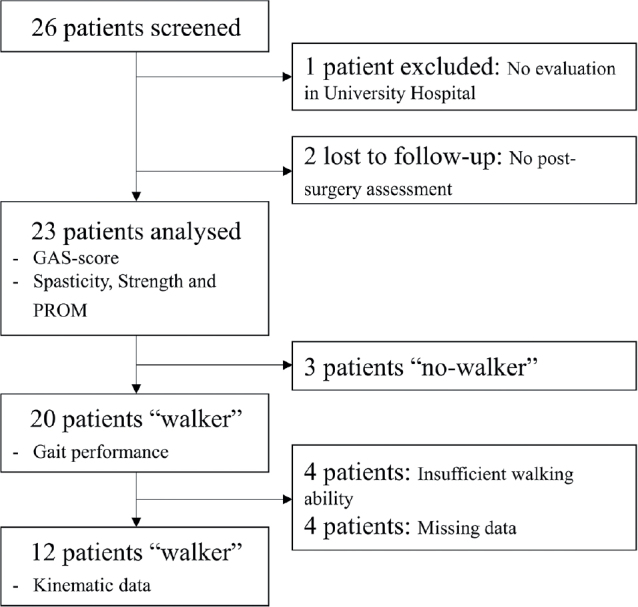
Study flow chart. GAS: Goal Attainment Scale ; PROM: Passive range of motion.

Twenty-six patients were screened; of these, 23 underwent pre- and post-operative assessment. Among the 20 ambulatory patients, 12 underwent 3D-IGA both before and after surgery (Fig. 2).

Surgery has been recommended to patients in various circumstances. Four patients underwent surgery after 1 or more phenolyses with waning effects. Three patients were offered surgery due to perceived inefficacy of BoNT injections, while 3 other patients experienced adverse events, such as weakness and asthenia from BoNT injections. Eleven patients were satisfied with the efficacy of BoNT, opted for surgery for various reasons: discomfort during injections, the necessity to renew treatment every 3–6 months, or the diversion of BoNT doses to other muscles. One patient underwent surgery as primary intention and another following a complication with his baclofen pump.

For preoperative assessment, all patients benefited from motor nerve block with a median (min–max) duration of 4.5 months before surgery (1–60). Ropivacaine was consistently used for all nerve blocks. Guidance was electrical, ultrasound or mixed, depending on the patient. Each block selectively targeted the anterior branch of the obturator nerve. In addition, for 3 patients, a posterior branch nerve block was administered as a secondary step, resulting in various outcomes: 1 patient experienced no change, another observed a deterioration in gait pattern, and the third showed improvement.

Demographic data are outlined in Table I. Only 2 patients had unilateral surgery, on the right side. The first was hemiplegic, while the second was tetraplegic due to a traumatic spinal injury and concurrently underwent neurotomy of the rectus femoris. Six patients underwent an associated AL tenotomy.

**Table I:** Patient characteristics (*n* = 23)

Characteristics	
Males, *n*	16
Diagnosis, *n*	
Cerebral palsy	11
Traumatic spinal cord injury	6
Multiple sclerosis or neuromyelitis optica	2
Syringomyelia	2
Hereditary spastic paraparesis	1
Cerebral palsy + multiple sclerosis	1
Deficit, *n*	
Paraplegia	14
Tetraplegia	8
Hemiplegia	1
Age at surgery, mean, years (min – max)	44 (18–73)
Time to follow-up after surgery, months, median (min – max)	29 (5–44)
mFAC (0 / 1–3 / 4–7 / 8), *n*	3 / 2 / 18 / 0

mFAC: modified Functional Ambulation Classification.

### Interventions between surgery and final assessment

The median duration between surgery and final assessment was 2 years and 5 months (Table I). Throughout this period, patients consistently participated in rehabilitation, with a frequency of at least 2 sessions per week, as outlined in Table SI. Some underwent other treatments that could influence their functional capacity. Notably, 3 patients continued BoNT injections in the adductor muscles post-surgery, while 10 patients received BoNT injections in other lower limb muscles. However, there is no available information regarding modifications to oral treatments for spasticity. One patient benefited from lumbar recalibration surgery.

### Goals and Goal Attainment Scale score

**Fig. 3:**
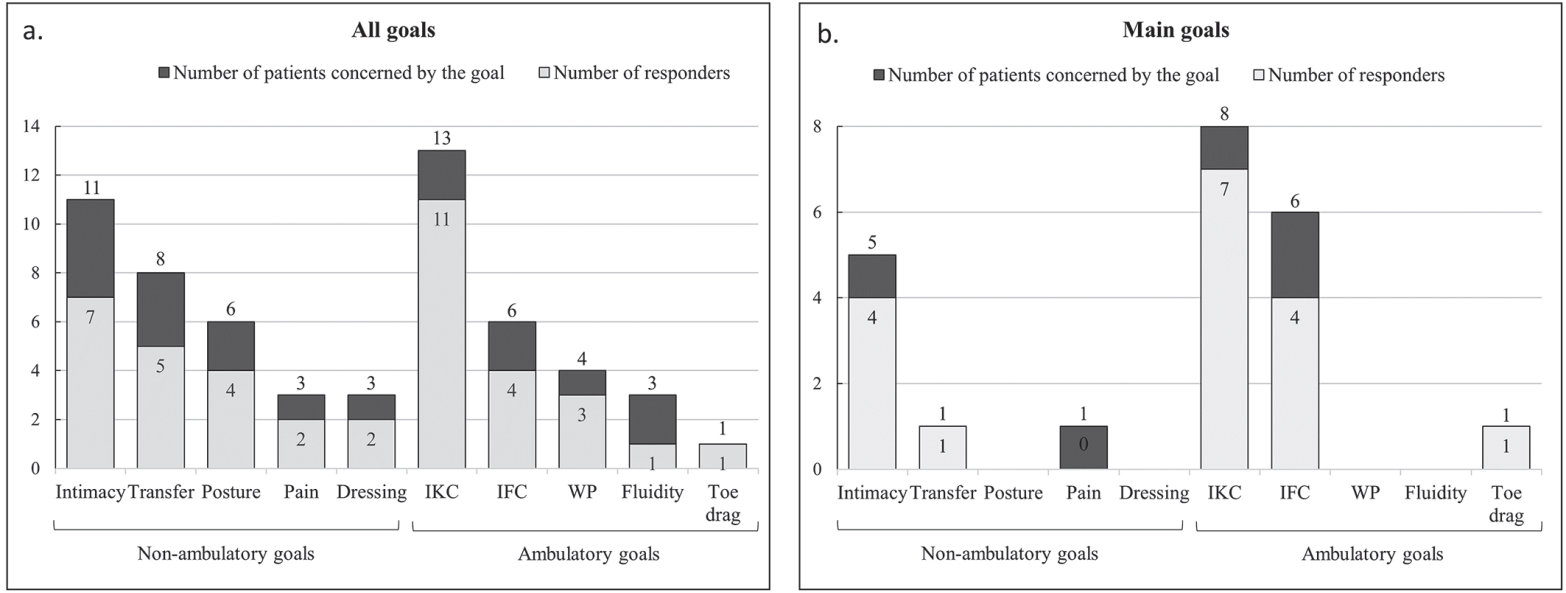
Goal categories and number of responders (Goal Attainment Scale (GAS) score ≥ 0).] IFC: inter-feet contact; IKC: inter-knee contact, WP: walking perimeter. Patients are considered responders if GAS score ≥ 0.

Ten categories of goals were identified (Fig. 3). Five were independent of ambulation and were “intimacy”, which included hygiene, intermittent catheterisation and sex, “transfer”, “pain”, “posture” and “dressing”. The remaining 5 were related to ambulation: “inter-knee contact”, “inter-feet contact”, “fluidity” (incorporating the subjective sensation of stability), “walking perimeter” (including fatigability), and “toe drag”. The number of goals per patient ranged from 1 to 5. The most common goals were inter-knee contact, intimacy and transfers, with 13, 11 and 8 patients prioritizing these, respectively (Fig. 3a). The only patient with the goal of reducing toe drag had a rectus femoris neurotomy associated with the ON. With the exception of fluidity, all goals had a success rate above 50%. Comparison of the rate of responders between non-ambulatory goals and ambulatory goals did not show a significant difference (65% vs 74%, respectively, *p* = 0.616). Among the 23 patients, 14 were responders to all of their goals, 4 to half of their goals, and 5 did not respond to any of their goals.

Fig. 3b details the main goals of each patient. For 1 patient, it was not possible to identify one main goal over another. Seventeen out of 22 patients (77%) were responders for their primary goals.

The 5 patients who were non-responders for their main goals also failed to achieve their secondary goals. Four of them reported an initial improvement that was not sustained over time (GAS score = –2 or –1) and the last 1 reported a worsening of his initial condition (GAS score = –3).

Within the cohort, 14 patients reported unexpected benefits, i.e. that were not originally designated as surgical goals. These unexpected effects could be linked to the previously mentioned categories (e.g. intimacy or pain) or unrelated (e.g. aesthetic improvement). Conversely, 1 patient reported a deleterious effect: more difficulty in getting out of bed. Details of the GAS scores for each goal and all unexpected effects are detailed in Table SII.

### Range of motion, spasticity and strength

A consistent improvement in extensibility was observed, either in passive abduction range or intercondylar distance (Table II). There was a significant decrease in spasticity in the 3 examination positions according to the MAS and to the MTS, indicating a delayed onset of catch during passive stretch in abduction.

**Table II:** Passive range of abduction, intercondylar distance and spasticity with the Modified Tardieu Scale (MTS) and the Modified Ashworth Scale (MAS)

	Pre, median (min; max)	Post, median (min; max)	*p*-value^a^
Passive range of abduction (°)			
Hip at 0° Knee at 0°, *n* = 29	30 (5;50)	30 (15;70)	< 0.001
Hip at 90° Knee at 90°, *n* = 29	40 (10;80)	50 (15;70)	0.077
Hip at 0° Knee at 90°, *n* = 23	30 (10;50)	45 (20;60)	< 0.001
Intercondylar distance (cm)			
Hip at 0° Knee at 0°, *n* = 13	30 (16;48)	32 (16;59)	0.183
Hip at 90° Knee at 90°, *n* = 15	35 (23;59)	45 (22;69)	0.009
Hip at 0° Knee at 90°, *n* = 1	38 (38;38)	40 (40;40)	NA
Spasticity (MTS)			
Hip at 0° Knee at 0°, *n* = 41	2 (0;2)	2 (0;2)	0.017
Hip at 90° Knee at 90°, *n* = 37	2 (1;2)	2 (0;2)	< 0.001
Hip at 0° Knee at 90°, *n* = 10	1.5 (1;2)	1 (0;2)	0.209
Spasticity (MAS)			
Hip at 0° Knee at 0°, *n* = 41	2 (0;5)	1 (0;2)	< 0.001
Hip at 90° Knee at 90°, *n* = 37	2 (1;4)	1 (0;2)	< 0.001
Hip at 0° Knee at 90°, *n* = 10	2.5 (1;4)	0.5 (0;2)	0.005

a Wilcoxon test for paired data.

NA: not applicable; *n*: number of limbs analysed (variability is due to missing values)

Regarding adduction strength, only post-operative data could be analysed, due to the limited availability of preoperative data. With the exception of 1 patient with syringomyelia and 1 patient with a complete spinal cord injury, all the patients retained an adduction strength of at least Held 3.

In terms of hip flexion strength, there was no significant difference between pre- and post-surgical values (median = 3 for both pre- and post-surgical; *p* = 0.227). Among the 15 patients assessed for hip flexion both before and after surgery, 2 exhibited loss of strength, transitioning from Held 4 to Held 3.

### Post-operative complications

Two post-operative complications were documented. One patient had post-operative sepsis, necessitating revision surgery. The second patient developed a local haematoma requiring surgical evacuation.

### Ambulatory capacity

Post-operative results on gait performance exhibited variability among patients (Table III). Specifically, 4 patients improved their mFAC score, while 2 patients experienced a decrease. Among these 2 patients, 1 patient had lost the capacity to walk; this patient had progressive pathology (neuromyelitis optica) and was already walking very precariously before the surgery. Two patients initiated use of a walking aid. The evolution of the walking perimeter was diverse, with 6 improving their perimeter distance while 5 experienced a decrease.

**Table III:** Difference between pre- and post-ambulatory capacity after surgery for each patient

	mFAC	WP, m (%)	Falls	Walking aids
*Progressive pathology*				
P6	–4	Ø	Ø	NA
P10	0	–100 (–29)	↗	↗
P13	0	0 (0)	=	=
P25	+1	–1400 (–47)	↗	=
*Non-progressive pathology*				
P3		+500 (+50)	=	=
P4	+1	+450 (+900)	=	↗
P5	0	+25 (+25)	=	=
P7	Ø	–100 (–25)	=	↘
P9	Ø	+5 (+100)	Ø	=
P11	0	–310 (–53)	=	↗
P12	–1	–80 (–62)	↘	=
P15	0	0 (0)	=	=
P16	0	Ø	=	=
P17	0	0%	=	=
P18	0	+500 (+100)	=	=
P19	+1	0 (0)	↘	=
P20	0	0 (0)	=	=
P22	+1	0 (0)	=	=
P24	0	+200 (+200)	↘	=
P26	0	0 (0)	=	=

WP: walking perimeter; mFAC: modified Functional Ambulation Classification; NA: not applicable; “Ø” represents missing data. Walking aid: 1 represents a unilateral aid (1 cane); 2 represents a bilateral aid (2 canes or 1 walker).

### Spatio-temporal analysis

**Fig. 4:**
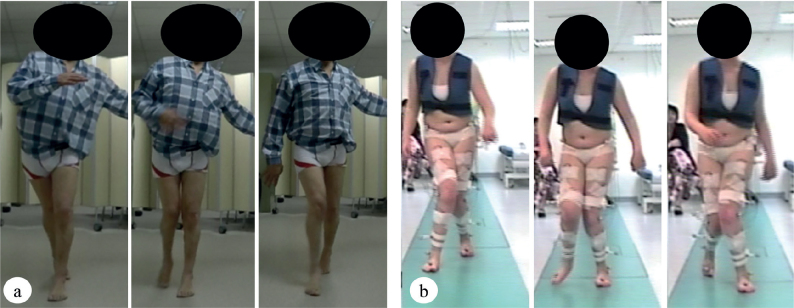
Two patients with inter-knee contact before the surgery. (a) Patient with an isolated hip adduction. The step width is reduced. (b) Patient with hip adduction associated with a pseudo-adduction (association of hip flexion and internal hip rotation) and valgus pronator foot. The step width is increased. We have obtained signed consent from the participants for the use of these images.

Before surgery, patients had a mean gait speed of 0.68 m/s. Five patients [P3; P10; P15; P17; P20] had a step-width increase exceeding 15 cm. In 4 cases [P3; P10; P15; P20], it was explained by the presence of a pseudo-adduction (association of hip internal rotation and hip flexion) with the adduction. This pseudoadduction often associated with a valgus hindfoot and arch collapse (Fig. 4).

Post-surgery, walking speed decreased in half of the patients and increased in only 2 patients, while step width increased beyond 2 SD for 3 patients (Table IV). Among these 3 patients, 2 had pseudo-adduction with increased step width preoperatively.

**Table IV:** Difference (95% confidence interval; 95% CI) of the kinematic and spatio-temporal data for each patient(after – before)

	Patient	Side	Speed (m/s)	Step width (cm)	Internal hip rotation:at IC	Internal FPA: at mid-stance	Clearance (mm)	Thigh flexion: max swing	Thigh abduction: max swing
Progressive pathology	P10	L	–0.29^†^* (–0.34; –0.25)	–0.51 (–1.87; 0.85)	–2.0 (–3.8; –0.1)	–5.0^a^* (–8.5; –1.4)	4.4 (–0.3; 9.0)	–0.9 (–2.0; 0.2)	–2.4^a^* (–3.4; –1.3)
		R			–12.4^a^* (–15.2; –9.6)	–1.4 (–4.2; 1.4)	–3.8 (–7.6; 0.6)	–3.9* (–4.8; –3.0)	1.1 (0.0; 2.1)
	P25	L^1;2^	0.01 (–0.04; 0.06)	2.21* (0.96; 3.46)	8.4^a^* (6.8; 10.1)	–0.1 (–2.5; 2.3)	0.3 (–5.4; 6.0)	–1.1* (–2.1; 0.0)	2.1^a^* (0.9; 3.3)
		R^1;2^			16.0^a^* (14.7; 17.3)	–2.2* (–4.1; –0.3)	1.0 (–4.5; 6.4)	1.4* (0.5; 2.3)	1.6* (0.1; 3.1)
Non-progressive pathology	P3	L	0.11* (0.05; 0.16)	8.15^†^* (6.84; 9.46)	8.6^a^* (5.9; 11.3)	5.1^a^* (1.5; 8.7)	7.3* (3.5; 11.1)	2.2* (1.0; 3.5)	–2.3^a^* (–4.5; –0.1)
		R^1^			–4.3* (–6.5; –2.2)	6.6^a^* (3.5; 9.7)	8.9* (2.8; 15.0)	4.4^a^* (3.4; 5.3)	2.9^a^* (0.9; 4.8)
	P4	L	–0.10^†^* (–0.14; –0.07)	7.38^†^* (5.87; 8.89)	–4.9* (–7.1; –2.7)	0.1 (–6.1; 6.2)	1.3 (–3.4; 6.1)	8.4^a^* (7.6; 9.2)	3.3^a^* (1.1; 5.4)
		R			–8.2^a^* (–1.09; –5.4)	3.6^a^ (–1.9; 9.1)	1.4 (–1.5; 4.4)	5.5^a^* (3.2; 7.8)	0.8 (–0.5; –2.2)
	P11	L	–0.24^†^* (–0.27; –0.21)	–0.33 (–1.45; 0.79)	–1.2 (–3.2; 0.9)	–6.2^a^* (–8.8; –3.5)	–6.6^†^* (–9.6; –3.6)	–1.5* (–2.3; –0.7)	4.5^a^* (3.5: 5.4)
		R^1;2^			0.7 (–1.4; 2.9)	–0.6 (–3.2; 2.0)	–1.6 (–4.0; 0.8)	–1.4* (–2.2; –0.6)	2.4^a^ (0.1; 4.6)
	P12	*L^¥^*	–0.11^†^* (–0.15; –0.07)	3.74* (1.56; 5.91)					
		R			1.2 (–0.3; 2.8)	–2.4 (–7.4; 2.7)	–3.3 (–9.8; 3.2)	2.9* (1.5; 4.4)	1.2 (–0.5; 2.9)
	P15	L	–0.20^†^* (–0.23; –0.17)	4.41^†^* (3.60; 5.22)	–1.2 (–2.8; 0.3)	2.3* (0.5; 4.1)	0.9 (–2.1; 4.0)	0.3 (–0.3; 0.9)	–4.0^a^* (–5.0; –3.1)
		R			5.8 (3.7; 8.0)*	–3.1^a^ (–6.3; 0.2)	–0.6 (–3.2; 2.0)	0.7 ( –0.2; 1.5)	–0.3 (–1.2; 0.7)
		R			9.2^a^* (7.4; 11.0)	–6.3^a^* (–8.9; –3.7)	–3.6* (–6.0; –1.3)	–7.7^a^* (–9.0; –6.4)	–0.2 (–1.2; 0.7)
	P18	L	–0.03 (–0.07; 0.00)	–1.65* (–2.3; –1.00)	7.6^a^* (6.1; 9.1)	–6.2^a^* (–7.9; –4.6)	2.9 (–1.1; 6.9)	2.8* (2.0; 3.6)	1.5* (0.9; 2.2)
		R			11.8^a^* (7.6; 16.1)	–1.9 (–4.0; 0.2)	0.2 (–2.9; 3.2)	2.1* (1.0; 3.2)	–0.3 (–1.2; 0.5)
	P20	L^1;2^	0.13^†^* (0.11; 0.16)	–0.58 (–1.74; 0.58)	4.2* (3.1; 5.2)	–2.3* (–4.0; –0.7)	11.2^†^* (8.4; 14.1)	5.3^a^* (4.6; 6.0)	–3.5^a^* (–4.9; –2.2)
		R			4.0* (2.7; 5.2)	–4.8^a^* (–6.5; –3.1)	–2.5 (–7.1; 2.2)	2.9* (2.3; 3.4)	1.1* (0.3; 2.0)
	P22	L	–0.02 (–0.06; 0.01)	2.45* (1.39; 3.52)	–1.1 (–2.6; 0.5)	0.4 (–1.4; 2.2)	1.4 (–1.8; 4.6)	1.8* (0.9; 2.6)	0.1 (–0.8; 1.0)
		R			6.2^a^* (4.4; 8.1)	–5.3^a^* (–7.2; –3.5)	–0.0 ( –3.2; 3.2)	–1.2* (–1.9; –0.5)	1.6* (0.6; 2.5)
	P24	L	0.12^†^* (0.09; 0.15)	3.85* (2.31; 5.39)	–16.6^a^* (–19.9; –13.4)	–14,3^a^* (–16.6; –12.0)	3.1* (0.9; 5.3)	2.7* (1.1; 4.2)	14.9^a^* (13.0; 16.8)
		R			–8.9^a^* (–10.4; –7.4)	–2.1 (–4.7; 0.5)	–2.1 (–6.7; 2.5)	3.2* (1.4; 4.9)	–3.2^a^ (–6.3; –0.1)

Max: maximum; L: Left limb; R: Right limb;

¥ non-operative limb;

FPA: Foot Progression angle.

1 Range of knee varus-valgus >12° before surgery;

2 Range of knee varus-valgus >12° after surgery.

* *p* < 0.05;

† difference >2 standard deviations(SD);

a Difference > minimal detectable change (4° for thigh flexion, 2° for thigh abduction, 3° for FPA, 6° for hip rotation). Cells are coloured when *p* < 0.05 and when difference > minimal detectable change or >2 SD (green for improvement, red for worsening).

### Kinematic data

In the 22 operated limbs, internal rotation of the foot decreased significantly (beyond the MDC) in 7 limbs and increased in only 2 limbs. For most patients, clearance remained unchanged. Proximally, kinematic modifications in the 3 planes (thigh flexion, thigh adduction, and hip rotation) varied among patients, sometimes yielding opposite results in the 2 limbs of the same patient (Table IV).

## DISCUSSION

Prior studies on ON have focused on its effect on impairments such as amplitude and spasticity. However, assessments of activity limitations were frequently lacking (22) or only briefly explored (18, 20, 23). One of the original features of this study is the emphasis on the reduction of activity limitations as the primary outcome, with the selection of individualized functional goals for each patient. Thus, 17 of 22 patients achieved their main goal and 14 of 23 patients achieved all their goals.

The GAS score was used as primary outcome, employing patient-reported measures, which are less reliable than objective measures. However, the advantage of these patient-reported measures is 2-fold: they are easy to use in daily practice and they allow a more ecological assessment (30). Secondarily, it is important to emphasize that this scale not only reflects the surgical impact on improving patient function, but also assesses the physician’s ability to define realistic goals. This process could have been facilitated by the motor nerve blocks systematically performed.

The failures to achieve some goals can be attributed, firstly, to challenges in defining objectives. To limit this risk, we systematically performed a motor block prior to surgery. This approach facilitates the establishment of realistic functional goals by simulating the effects of surgery over a few hours (31, 32). Motor blocks help distinguish between muscular overactivity and contracture (26, 33) and differentiate the contribution of muscles such as the AL, AB and gracilis from those of the AM in causing patient discomfort. Thus, this risk is more pronounced when assessing subjective dimensions such as fluidity.

Failures can also be explained by the underlying pathophysiology of muscle overactivity, encompassing spasticity, spastic dystonia and co-contraction (1). Neurotomy disrupts the myotatic reflex by severing afferent fibres, leading to a reduction in spasticity as evidenced in this study and others (24). While sensory reinnervation is theoretically improbable, explaining the long-term effectiveness of neurotomy on spasticity (16), its impact on spastic dystonia and co-contraction is less certain. In 1966, Denny-Brown (34) demonstrated the persistence of spastic dystonia despite the afferent blockade at the medullary level. Therefore, the efficacy of neurotomy on dystonia probably stems from partial motor neurone sectioning. Unlike sensory fibres, motor reinnervation of muscles can occur through the sprouting phenomenon of intact motor neurones (16), potentially allowing for the recurrence of spastic dystonia and co-contraction alongside motor reinnervation.

Despite a 74% achievement rate for walking goals, the results regarding objective ambulatory capacity and walking patterns exhibited variability. On the one hand, 3 patients decreased their fall frequency, and step width increased for 3 of the 12 patients, consistent with observations following chemodenervation with phenol (11). On the other hand, 2 patients started to use walking aids, and walking speed decreased in half of the patients, contrary to the results seen after BoNT injections (9). Of these patients, 1 had cerebral palsy associated with multiple sclerosis, 4 had cerebral palsy, and 1 had a spinal cord injury. These findings may be explained by the natural progression of the disease, especially considering the potentially significant interval between the 2 assessments, sometimes exceeding 6 years. While cerebral palsy is not typically considered a progressive condition, studies indicates a decline in gait function over time (35). Results regarding walking perimeter are variable, and caution is advised in interpretation due to the oral collection of the results without objective recording.

The contribution of 3D-IGA in assessing the effects on adduction is limited, primarily due to unreliable kinematic data in the frontal plane, attributed to a large margin of error (29). No significant kinematic changes were observed in the sagittal plane, except for 1 patient who exhibited reduced hip flexion during swing, possibly due to diminished hip flexor strength, although hip flexion strength seemed preserved in supine position. The action of adductor muscles in the transverse plane remains a topic of debate. Lacote considers all adductor muscles (excluding the posterior head of the AM) as external rotators (36), while Neumann (37) suggests that the AL and AB act as internal rotators. The lack of consensus is influenced by variations in muscle action related to hip position. For instance, Arnold’s computer model (38) demonstrated that an internally rotated hip position increases the moment arm of internal rotation of the proximal adductors (AL and AB) and the moment arm of external rotation of the gracilis. Excessive femoral anteversion, common in cerebral palsy patients further influences adductor muscles action in the horizontal plane, with studies indicating reduced participation of adductor muscles in internal rotation (39). In addition, the adductor muscles’ vertical orientation imparts relatively low torque in the horizontal plane. In the current study, among the 22 analysed limbs, 4 showed decreased hip internal rotation while 7 exhibited increased their hip internal rotation after ON. However, analysing the foot progression angle, strongly influenced by hip rotation, revealed that 7 limbs decreased their internal rotation, with only 2 increasing it. This disparity may stem from a lack of reproducibility due to the lower performance of 3D-IGA in analysing hip rotation movements (29). Notably, 4 patients displayed a knee valgus-varus range greater than 12°, indicative of a poorly defined knee flexion-extension axis that could distort hip rotation values (40, 41). This substantial disparity in results in the horizontal plane illustrates the challenge of defining adductor muscles action in the horizontal plane. Thus, ON should not be performed with the objective of modifying hip rotation. Therefore, distinguishing between patients with adduction and those with pseudo-adduction, a combination of hip flexion and internal rotation, is imperative. Both deformities may be responsible for inter-knee contact, but step width may be increased in cases of pseudo-adduction. In this study, 4 patients present inter-knee contact due to pseudo-adduction associated with hip adduction. Two of them were non-responders for all ambulatory goals; the poor results for these 2 patients could be explained by a predominant pseudo-adduction compared with adduction.

Another study investigated kinematic changes in walking following ON, but the population and surgical project were different (24). This study focused exclusively on patients with spinal cord injury exhibiting severe spasticity, of which 8 were ambulatory (walking) patients. Furthermore, ON was consistently paired with a neurotomy of the tibial nerve and occasionally of the hamstring branches of sciatic nerve. The results are consistent with our own, with no significant changes in gait speed, step width or maximum hip flexion. There was no analysis in the frontal plane.

In terms of safety, the surgical procedure is performed in the inguinal fold, posing a potential risk of infection. Indeed, 1 patient presented post-operative sepsis. Another patient had a post-operative haematoma. These 2 events required surgical revision. In term of functionality, only 1 patient reported a deleterious effect in getting up from the supine position.

This study has several limitations owing to its retrospective design. First, the GAS methodology could not be applied perfectly. For the majority of patients, only the score “0” was defined before surgery. Therefore, the score “+2” had to be chosen after surgery. Then, the assessment of the walking perimeter is limited by its subjective nature. A 6-min walk test (6MWT) would have been more objective and reproducible.

The small and heterogeneous population size in this study precluded the examination of results in subpopulation.

In addition, the interval between pre- and post-operative 3D-IGA was variable, sometimes exceeding 6 years. During this period, factors other than the surgery could impact patients’ abilities, including the use of other therapeutics, disease progression, ageing, and weight gain. The primary aim of this study was to assess patients’ achievement of functional goals using the GAS scale. Although kinematic data were studied in parallel, interpretation was challenging due to the time gap and variable marker position between the 2 3D-IGA sessions. Conducting a 3D-IGA before and after motor nerve block, while maintaining consistent marker positions, would enhance the understanding of the functional role of adductor muscles and the consequences of their weakening.

This study discussed the efficacy of neurotomy on the 3 components of spastic overactivity. Including information on patients’ spontaneous positions, active range of motion, and EMG analysis during rest and active movements would offer a more comprehensive understanding of the impact of neurotomy on spastic dystonia and co-contraction, relying solely on data (42).

In conclusion, the majority of patients achieved their goals following surgery. We recommend conducting a pre-surgery motor nerve block to determine the potential benefits of surgery of the hip adductor muscles and to establish relevant goals with the patient. The results were broadly similar for ambulatory and non-ambulatory goals. However, for ambulatory goals, surgery of the hip adductor muscles may not be effective in patients with isolated pseudo-adduction. Caution is advised in patients with inter-condylar contact and increased step width. Future studies with a larger population are necessary to validate these findings and to better define prognostic factors.

## Supplementary Material


