# Supplementary material for: FUNCTIONAL OUTCOMES FOLLOWING SURGERY FOR SPASTIC HIP ADDUCTOR MUSCLES IN AMBULATORY AND NON-AMBULATORY ADULTS
Source: J Rehabil Med. 2024 Mar 22;56:18356. doi: 10.2340/jrm.v56.18356 (PMC10985928; doi:10.2340/jrm.v56.18356)
Supplement: Supplementary file 1 [file JRM-56-18356-s1.pdf]

Supplementary material has been published as submitted. It has not been copyedited, typeset or checked for scientific content by Journal of Rehabilitation Medicine

Table SI. Type of surgery and type of rehabilitation post-surgery

Surgery		Rehabilitation	
**Side of neurotomy, n**		**Place, n**	
Left	21	home	1
Right	23	physiotherapist's office	12
		Hospital day care	2
		Full hospitalisation	8
**Tenotomy, (Left, right or both), n**		**Duration, n**	
Yes	6	< 1 month	2
No	17	1 to 3 months	11
		> 3months	9
		Not reported	1
**Other neurotomy, n**		**Intensity, n**	
Yes	1	1 per week	0
No	22	2 or 3 per week	12
		> 3 per week	11

Table SII. GAS-Score, Unexpected effects and global satisfaction

	Patient	GAS score				Goals achieved	Unexpected effects (subjective assessment)		Global satisfaction
		Main goal		Other goals					
No Walker	P2	Transfer	2	Intimacy<br>Posture	2<br>0	100%			Ø
	P21	Intimacy	0	Transfer	-1	50%	+<br>+	↘ Clonus in left lower limb<br>↗ Dressing	60%
	P23	Intimacy	1	Transfer	2	100%			100%
Walker	P3	Intimacy	2	Fluidity	-1	50%			100%
	P4	IFC	2	WP<br>Intimacy<br>Transfer<br>Dressing	0<br>0<br>1<br>1	100%	+<br>+<br>+<br>+<br>+	↘ IKC (60%)<br>↘ Toe drag (50%)<br>↗ Intimacy hygiene (90%)<br>↗ Posture in the chair (90%)<br>Stairs without human assistance	70%
	P5	IKC	0			100%	+<br>+<br>+	↗ Fluidity (40%)<br>↗ WP<br>↗ Intimacy hygiene (50%)	Ø
	P6	Pain	-2	Intimacy	-2	0%	+	Withdrawal of abduction pad in chair	100%
	P7	IKC	2	WP	0	100%	+<br>+<br>+<br>+<br>+	Only 1 cane for short distance<br>↗Posture in chair (80%)<br>↗ Aesthetic (rated at 80%)<br>↘ Low back pain<br>↗ For sexual intercourse	75%
	P9	Ø		IKC<br>Intimacy<br>Transfer<br>Dressing	0<br>-1<br>0<br>-1	50%			80%
	P10	IKC	2	WP	0	100%	+<br>+<br>+<br>+<br>+	↘ Toe drag<br>↘ Pain (100%)<br>↗ Putting on socks/shoes<br>↘ IFC<br>↗Intimacy hygiene (85%)	100%
	P11	IKC	0			100%	+	↗ Intimacy hygiene (60%)	60%
	P12	IFC	-2	Intimacy<br>Posture	-2<br>-2	0%	+	↗ Dressing	0%
	P13	IKC	0	Intimacy<br>Transfer<br>Posture	2<br>2<br>2	100%	+<br>-	↗ Fluidity<br>Difficulty getting out of bed	Ø
	P15	IFC	1	IKC	2	100%	+<br>+	↘ IFC<br>↗ Fluidity (30%)	70%
	P16	Intimacy	0	Transfer<br>Posture<br>Dressing	-2<br>-2<br>0	50%	+	↗ Dressing	80%
	P17	Toe drag	0	IKC<br>Pain<br>Posture	0<br>0<br>0	100%			Ø
	P18	IKC	0	Pain	0	100%	+	↘ Toe drag with ↘ falls	80%
	P19	Intimacy	-3	IKC<br>Transfer	-2<br>-2	0%	+	↘ Falls	50%
	P20	IKC	-1	Fluidity<br>WP	-1<br>-2	0%			50%
	P22	IKC	1			100%			Ø
	P24	IFC	0	Fluidity	2	100%			Ø
	P25	IFC	2	IKC<br>Posture	0<br>0	100%	+	↗ Transfer into car	70%
	P26	IFC	-2			0%			Ø

IFC: inter-feet contact; IKC: inter-knee contact; WP: walking perimeter.
"+" indicates an unexpected beneficial effect, while the "-" indicates a harmful effect. "Ø" represents missing data.
